# Applying artificial intelligence to disease staging: Deep learning for improved staging of diabetic retinopathy

**DOI:** 10.1371/journal.pone.0179790

**Published:** 2017-06-22

**Authors:** Hidenori Takahashi, Hironobu Tampo, Yusuke Arai, Yuji Inoue, Hidetoshi Kawashima

**Affiliations:** Department of Ophthalmology, Jichi Medical University, 3311–1 Yakushiji, Shimotsuke-shi, Tochigi, Japan; International University of Health and Welfare, JAPAN

## Abstract

**Purpose:**

Disease staging involves the assessment of disease severity or progression and is used for treatment selection. In diabetic retinopathy, disease staging using a wide area is more desirable than that using a limited area. We investigated if deep learning artificial intelligence (AI) could be used to grade diabetic retinopathy and determine treatment and prognosis.

**Methods:**

The retrospective study analyzed 9,939 posterior pole photographs of 2,740 patients with diabetes. Nonmydriatic 45° field color fundus photographs were taken of four fields in each eye annually at Jichi Medical University between May 2011 and June 2015. A modified fully randomly initialized GoogLeNet deep learning neural network was trained on 95% of the photographs using manual modified Davis grading of three additional adjacent photographs. We graded 4,709 of the 9,939 posterior pole fundus photographs using real prognoses. In addition, 95% of the photographs were learned by the modified GoogLeNet. Main outcome measures were prevalence and bias-adjusted Fleiss’ kappa (PABAK) of AI staging of the remaining 5% of the photographs.

**Results:**

The PABAK to modified Davis grading was 0.64 (accuracy, 81%; correct answer in 402 of 496 photographs). The PABAK to real prognosis grading was 0.37 (accuracy, 96%).

**Conclusions:**

We propose a novel AI disease-staging system for grading diabetic retinopathy that involves a retinal area not typically visualized on fundoscopy and another AI that directly suggests treatments and determines prognoses.

## Introduction

Disease staging involves grading severity or progression of illness. The purpose of disease staging is to improve the accuracy of treatment decisions and prognosis prediction. To ensure reproducibility, disease staging is done by using clear, verbalizable observations. In addition, skilled physicians gather impressions from patients’ non-verbalizable or unclear observations. However, disease staging has greater accuracy than impressions[1] because of human inconsistencies[2–4] caused by the exhaustion[5,6] or blood-sugar[7,8] levels of the physicians. However, this is not the case with AI.

Deep learning, a branch of the evolving field of machine learning, has advanced greatly in recent years. In 2012, a deep convolutional neural network, AlexNet,[9] showed increased accuracy in the classification of high-resolution images. In 2014 and 2015, similar versions, including Google’s deep convolutional neural network GoogLeNet[10] and Microsoft’s deep convolutional neural network ResNet,[11] each exceeded the human limit of accuracy in image recognition.

**Fig 1:**
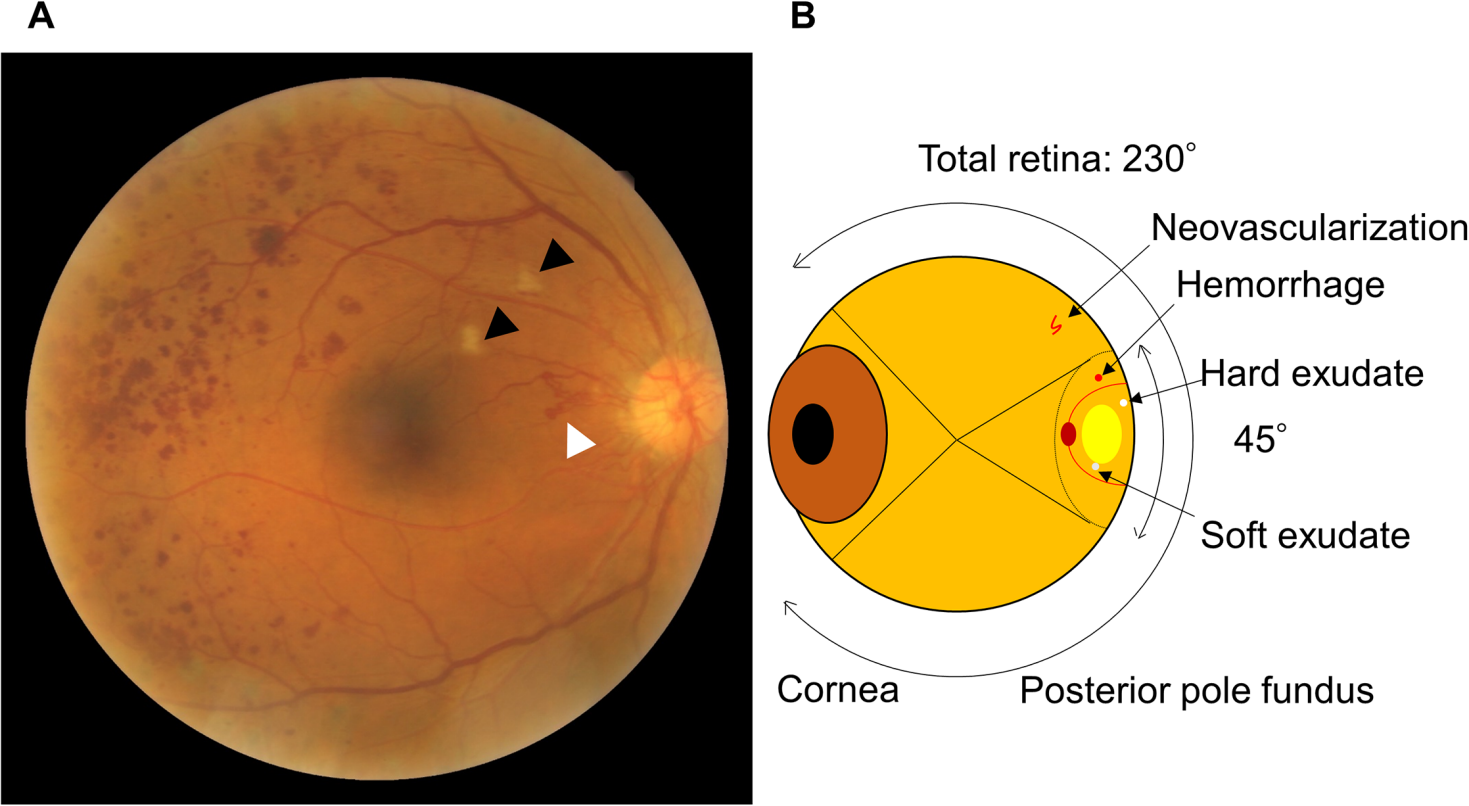
A fundus photograph and schema of an eye ball. **(**A) Posterior pole fundus photograph with proliferative diabetic retinopathy. White arrow head: Neovascularization. Black arrow heads: Soft exudates. Red dots: hemorrhage. (B) Schema of an eye ball. Because there is neovascularization, this eye requires panretinal photocoagulation. Because the neovascularization is not shown in a normal fundus camera field, it could be misdiagnosed as simple diabetic retinopathy, which does not require any therapy.

Diabetic retinopathy is the leading cause of blindness worldwide.[12] Davis staging[13] is one common staging method for diabetic retinopathy. In our practice, we use the modified Davis staging (Table 1). Since diabetic retinopathy progresses from simple diabetic retinopathy (SDR) to pre-proliferative retinopathy (PPDR) to proliferative diabetic retinopathy (PDR), it is necessary to perform ocular panretinal photocoagulation in PDR (Fig 1A).

**Table 1:** Modified Davis grading.

Grading	
SDR	Microaneurysm, retinal hemorrhage, hard exudate, retinal edema, and more than 3 small soft exudates
PPDR	Soft exudate, varicose veins, intraretinal microvascular abnormality, and non-perfusion area over one disc area
PDR	Neovascularization, pre-retinal hemorrhage, vitreous hemorrhage, fibrovascular proliferative membrane, and tractional retinal detachment

SDR, simple diabetic retinopathy; PPDR, pre-proliferative diabetic retinopathy; PDR, proliferative diabetic retinopathy.

A fundus photograph is usually taken 45° to the posterior pole of the fundus. This only shows the most disease-prone areas, whereas the entire retina can be viewed at an angle of 230°. Therefore, grading systems using only one photograph may categorize a PDR patient as having SDR since neovascularization or other PDR signs are outside the 45° angle to the posterior pole of the fundus (Fig 1B). Thus, a single photograph is not suitable for staging diabetic retinopathy; it is only useful for detecting the presence of diabetic retinopathy.[14] A more appropriate grading of diabetic retinopathy involves fluorescein angiography with nine photographs or ultra-widefield photography. Four[15] or more[16] photographs are required to screen for diabetic retinopathy.[14] Recently, ultra-widefield scanning laser ophthalmoscopy was introduced. This method allows up to 200° imaging of the retina in a single image.[17] However, the use of four or more conventional photographs and ultra-widefield ophthalmoscopy is more complicated than a single conventional photograph and is not applicable to current screening methods.

Skilled physicians infer strongly negative impressions from some single SDR conventional photographs—when some features of PDR are predicted to be outside a 45° angle to the posterior pole, indicating poor prognosis—and weaker impressions from other SDR single conventional photographs—when no features of PDR are predicted to be outside the 45° angle, indicating good prognosis. Adopting this criterion, deep learning increases the possibility of identifying neovascularization or other features of PDR outside a 45° angle to the posterior pole by detecting non-verbalizable unclear signals.

Here, we show an AI that grades diabetic retinopathy involving a retinal area that is not normally visible on fundus photography using non-verbalizable features that resemble impressions. The AI has greater accuracy than conventional staging and can suggest treatments and predict prognoses.

Our proposed AI disease-staging system can recommend treatments or determine prognoses from both verbalizable and non-verbalizable observations. We believe that this staging system will promote disease staging by helping to improve disease outcomes.

## Methods

### Study design

This single-site, retrospective, exploratory study was performed in an institutional setting.

### IRB approval

Institutional review board approval was obtained. Informed consent was obtained from all subjects. The protocol adhered to the tenets of the Declaration of Helsinki.

### Materials

We obtained 9,939 posterior pole photographs from 2,740 patients with diabetes. Nonmydriatic 45° field color fundus photographs were taken of four fields in each eye annually at Jichi Medical University between May 2011 and June 2015 on between one and four separate occasions.

Color fundus photographs of four fields at 45° were obtained using a fundus camera (AFC-230; NIDEK Co., Ltd., Aichi, Japan). Color fundus photographs of either one or four fields were graded by modified Davis grading (Table 1). Of the photographs, 6,129 were NDR, 2,260 were SDR, 704 were PPDR, and 846 were PDR. The grader was not told that the grading would be used in this study. The original photographs were 2,720 × 2,720 pixels. The outlying 88 pixels of the margin were deleted and the photographs shrunken by 50% to 1,272 × 1,272 pixels to fit in the graphical processing unit memory (12 GB, GeForce GTX TITAN X; NVIDIA Co., Santa Clara, CA, USA). Four graphical processing units were used simultaneously. The modified GoogLeNet was used in an open framework for deep learning (Caffe, Berkeley Vision and Learning Center, Berkeley, CA, USA). The neural networks were trained for 400 epochs.

The accuracy (sensitivity) value of the prediction itself is not useful due to variations in the numbers in each group. The accuracy value tends to be high and is meaningful in only comparisons between AIs or graders. Because the normal Fleiss' kappa value also has little value due to variations in the numbers in each group, the prevalence- and bias-adjusted kappa (PABAK)[18–20] was calculated as a main outcome instead. On the other hand, statistical comparison between PABAK values is difficult. Statistical analysis was performed using JMP Pro software version 12.2.0 (SAS Institute, Cary, NC, USA).

### Grading including unseen areas

**Fig 2:**
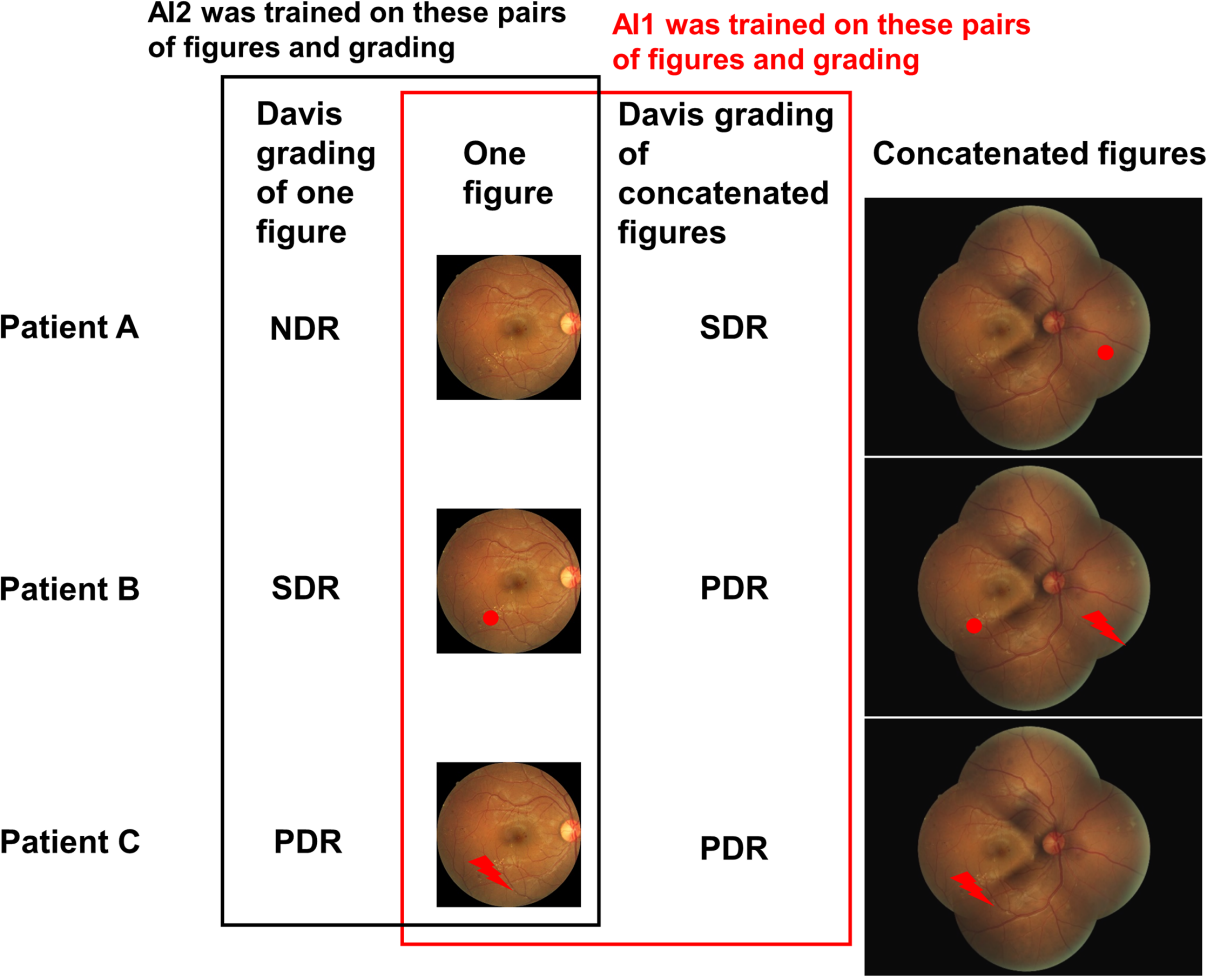
Two training methods and a three-patient model. AI1 was trained on the pairs of one figure and the modified Davis grading of a concatenated figure. AI2 was simply trained on the pairs of one figure and the modified Davis grading of this figure. Red dot: dot hemorrhage representing SDR. Red lightning bolt: neovascularization representing PDR.

We trained a modified GoogLeNet deep convolutional neural network with 9,443 45° posterior pole color fundus photographs using manual staging with three additional color photographs (AI1; Fig 2). We also trained the neural network with the same photographs using manual staging with only one original photograph (AI2; Fig 2). To maximize training sets, only 496 of the 9,939 photographs were randomly chosen (5%) for cross-validation three times from the eyes that were photographed only once. The remaining 9,443 photographs were used for training and, instead of a small validation set, the trained network that had the intermediate accuracy of the three networks was chosen. The following GoogLeNet modifications were applied: deletion of the top 5 accuracy layers, expansion of crop size to 1,272 pixels, and reduction of batch size to 4. The base learning rate was 0.001. To promote robustness of the mean accuracy of AI1, *K*-fold cross-validation (*K* = 20) was performed. To compare with the results for another neural network, AI1 was trained with the ResNet^11^ model.

In 20 randomly chosen PDR validation images, we checked what characteristics were used by AI1 in images of the middle layer.

### Grading using actual prognoses

We graded 4,709 of the 9,939 posterior pole fundus photographs using real prognoses (Table 2). The remaining 5,230 photographs were excluded because the patients only visited the clinic once. Patients with SDR and higher staging were recommended for a second medical checkup. They underwent pan-fundus ophthalmoscopy. Patients with suspected PDR also underwent fluorescein angiography while those with PDR received panretinal photocoagulation. Eyes with PDR and vitreous hemorrhage, fibrovascular proliferative membrane, or tractional retinal detachment underwent vitrectomy. Eyes with diabetic macular edema received anti-vascular endothelial growth factor therapy,[21] local steroid therapy,[22] or focal photocoagulation.[23] Subsequent fundus photographs were taken between 6 months and 2 years. The remaining visual acuity represents visual prognosis within 0.2 logMAR after 6 months.

**Table 2:** Grading by actual prognosis.

Grading	Needed treatments	When	Prognosis	No. of 4709	NDR	SDR	PPDR	PDR
0	None	Next visit	All	4445	2479	1289	333	344
1	DME treatments			12	0	5	1	6
2	PRP		Improve	20	1	2	14	3
3			Stable	6	1	1	0	4
4			Worsen	0	0	0	0	0
5	Vitrectomy		Improve	2	0	0	0	2
6			Stable	4	0	0	0	4
7			Worsen	2	0	0	0	2
8	DME treatments	Current visit	All	16	0	0	7	9
9	PRP		Improve	108	0	0	40	68
10			Stable	31	0	1	5	25
11			Worsen	29	0	0	9	20
12	Vitrectomy		Improve	10	3	0	0	7
13			Stable	16	0	0	1	15
14			Worsen	8	0	0	0	8

DME, diabetic macular edema; PRP, panretinal photocoagulation.

Among the 4,709 photographs, 95% were used to train the neural network and 5% were used for validation; these photographs were randomly chosen from all grades at equal rates. The training and validation sets were selected three times and the trained network that had the intermediate accuracy of the three networks was chosen. GoogLeNet modifications included expansion of the crop size to 1,272 pixels and reduction of the batch size to 4. The base learning rate was 0.0001.

Prediction rates using previous grading were calculated as follows. First, each actual prognosis staging ratio in NDR, SDR, PPDR, and PDR was calculated in 95% of the 4,709 photographs, which were the same as the photographs used for training the neural networks. Second, the prediction ratio of the real prognosis staging was calculated in each of the 5% of the 4,709 photographs, which were also the same photographs used for validating the neural networks.

We had three retinal specialists. HT, the first author, is a 15th year ophthalmologist and has performed 200 vitrectomies per year. YA is a 5th year ophthalmologist and has performed 200 vitrectomies per year. YI, a co-author, is a 17th year ophthalmologist and has performed 100 vitrectomies per year. All specialists blindly graded the same 5% of all 4,709 photographs to 0–14.

## Results

### Grading including unseen retinal areas

We trained the modified GoogLeNet deep convolutional neural network with 9,443 45° posterior pole color fundus photographs using manual grading in three additional color photographs for each initial photograph. The PABAK of the trained network was 0.74 (correct answer with the maximum probability in 402 of 496 photographs; mean accuracy, 81%). Similar training using manual grading with one photograph achieved a PABAK of 0.71 (correct answer with the maximum probability in 381 of 496 photographs; mean accuracy, 77%) but 0.64 (correct answer with the maximum probability in 362 of 496 photographs; mean accuracy, 72%) with grading of four photographs. The accuracy for the four photographs trained by one photograph was significantly lower than for the four photographs trained by four photographs (*P* < 0.0001, two-sided paired *t*-test). The mean accuracy of *K*-fold cross-validation (*K* = 20) was 0.80.

ResNet with 1,272 pixels could not be used on a TITAN X with 12GB memory because there was not enough memory. The maximum sizes that could be trained were 636 pixels for ResNet-52 and 590 pixels for ResNet-152. The mean accuracy was 0.62 for each, which is significantly worse than seen using GoogLeNet with 1,272 pixels (*P* < 0.0001, two-sided paired *t*-test).

**Fig 3:**
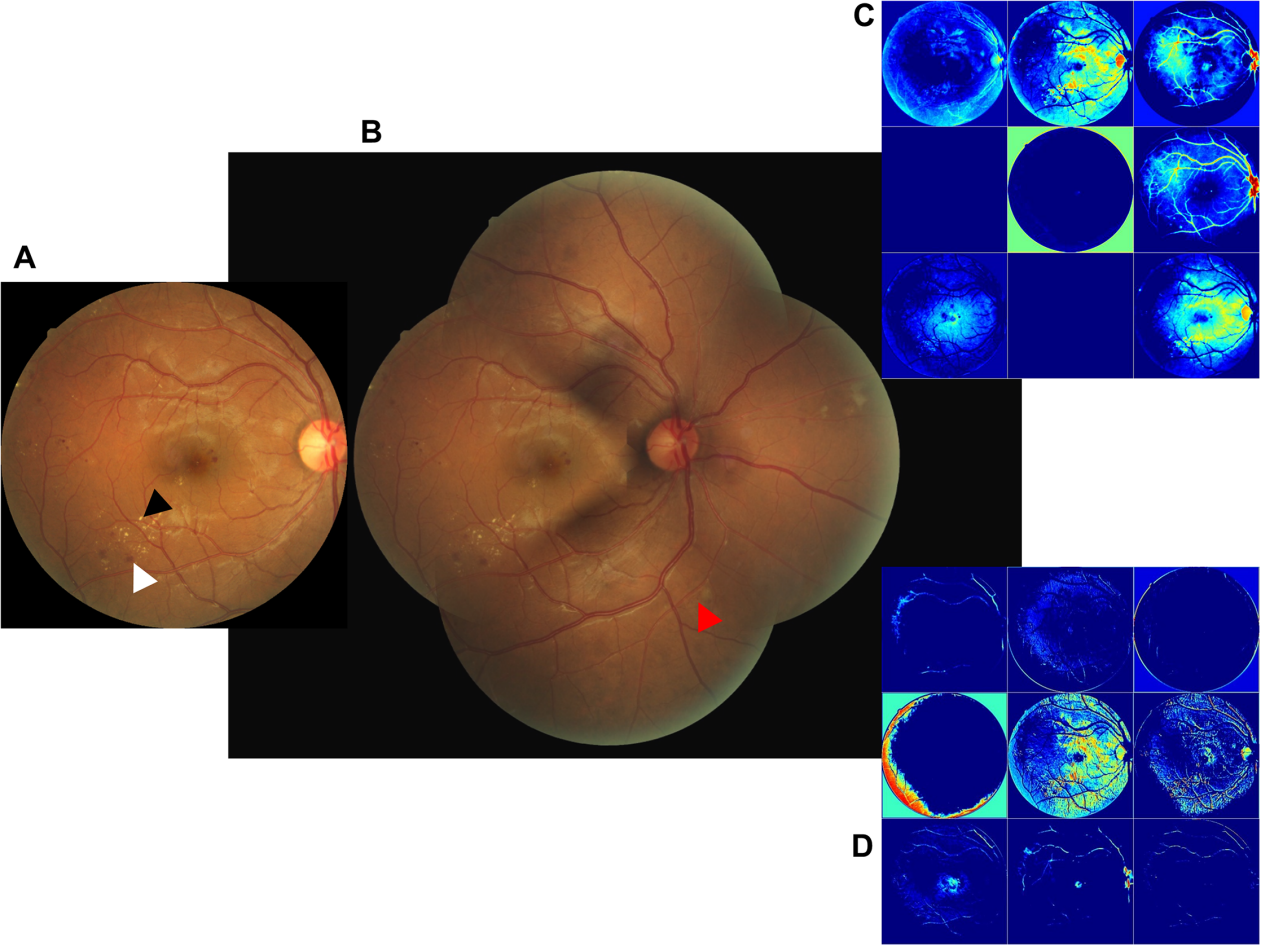
The representative fundus photograph which was graded as SDR in one photograph but PDR in four photographs. **(**A) Horizontal inversion left fundus photograph of DMR showing hemorrhage (white arrow head) and hard exudates (black arrow head) without neovascularization or vitreous hemorrhage. Classification is SDR. (B) Horizontal inversion, composite of four left fundus images showing neovascularization (red arrow head). Classification is PDR. (C) Visualization of the conv2/norm2 layer of the four photograph-trained GoogLeNet. (D) Visualization of the conv2/norm2 layer of GoogLeNet trained by one photograph. Trained network suggested NDR 1% and SDR 99%. Blurry view by the four photograph-trained neural networks; distinct view by the one photograph-trained neural network.

The representative fundus photograph was graded as SDR in one photograph (Fig 3A) but PDR in four photographs (Fig 3B). We compared the visualization of the conv2/norm2 layer of GoogLeNet that was trained by either one or four photographs. One photograph-trained neural networks visualized the photograph with higher frequency than the four photograph-trained neural networks (Fig 3C and 3D). A one photograph-trained neural network could detect small retinal hemorrhages and hard exudates. Here, the four photograph-trained neural networks suggested NDR, SDR, PPDR, and PDR values with 28%, 26%, 23%, and 23% likelihood values, respectively. The one photograph-trained neural networks suggested NDR and SDR with 1% and 99% likelihood, respectively. Thus, the four photograph-trained neural networks are more useful than one photograph-trained neural networks and are able to grade diabetic retinopathy involving a retinal area that is not visualized on one photograph.

**Fig 4:**
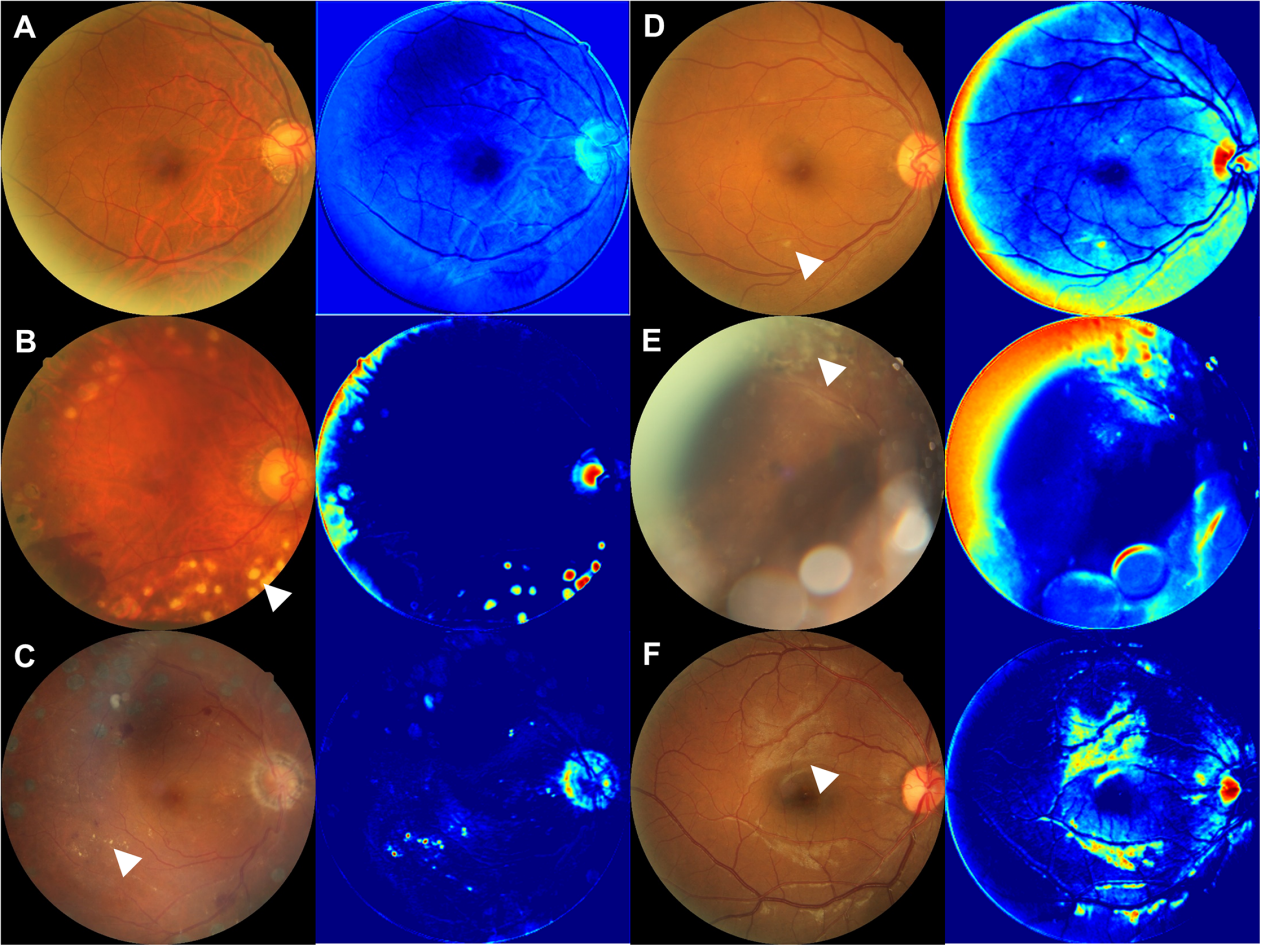
The images from the middle layer of the neural network. **(**A) Representative color fundus photograph of NDR and an image of the middle layer, which has few characteristics. B-F: Representative color fundus photographs of PDR and their images of the middle layer. (B) Laser scars (white arrow head) were enhanced in the middle image. (C) Hard exudates (white arrow head) were enhanced in the middle image. (D) Soft exudates (white arrow head) were enhanced in the middle image. (E) Proliferative membranes (white arrow head) were enhanced in the middle image. (F) Reflections of the retina (white arrow head) were enhanced in the middle image.

The images from the middle layer suggested several characteristics that were used by AI1. A photocoagulation scar was seen in 7 of the 20 randomly chosen PDR validation images (Fig 4B), indicating PDR treatment; hard exudate in 7 of the 20 (Fig 4C), a criterion of SDR; soft exudate in 2 of the 20 (Fig 4D), a criterion of SDR; proliferative membrane in 2 of the 20 (Fig 4E), a criterion of PDR; and surface reflection of the retina in 2 of the 20 (Fig 4F), which was not used as a criterion of diabetic retinopathy. The images with NDR had few characteristics (Fig 4A).

The AI1 is demonstrated on our website at http://deepeyevision.com.

### Grading using actual prognosis

A total of 4,709 posterior pole color fundus photographs were graded to 0–14. The grading criteria were as follows: “not requiring treatment”, “requiring treatment at the next visit,” or “requiring treatment in the current visit”. The treatment required was graded as “treatment for diabetic macular edema”, “panretinal photocoagulation”, or “vitrectomy”. Visual acuity was “improved”, “stable”, or “worsened” (Table 2).

The modified GoogLeNet was trained using 95% of the graded photographs. The PABAK of the trained neural network was 0.98 (mean accuracy, 96%) in the 224 photographs that were not used in the training phase. The PABAK of the traditional modified Davis staging was 0.98 (mean accuracy, 92%) in the same 224 photographs. The three retinal specialists (HT, YA, and YI) had PABAK values of 0.93 (mean accuracy, 93%), 0.92 (mean accuracy, 92%), and 0.93 (mean accuracy, 93%), respectively. The trained neural network was significantly more precise and consistent than the traditional grading (overall *P* < 0.0001): HT (*P* = 0.018), YA (*P* = 0.0067), and YI (*P* = 0.034) (two-sided paired *t*-test).

The false negative rate—when the grade was “not requiring treatment” but treatment was actually needed—was 12%. The false positive rate—when the grade was “requiring treatment in the current visit” but treatment was actually not needed at the next visit—was 65%.

## Discussion

Although diabetic retinopathy is the main target of machine learning, many challenges remain.[24] Nonetheless, some machine learning approaches have been used, such as that of ter Haar Romeny et al.,[25] who reported brain-inspired algorithms for retinal image analysis, which is a sophisticated approach. In our study, we simply used a convolutional neural network for general image classification. However, we used classification information from unseen areas as training data for the deep learning, achieving a PABAK of 0.74 and an accuracy of 81%. Using very deep convolutional neural network, Xu et al.[26] reported an accuracy of 94.54%. This accuracy is higher than ours, but the advantage of our trained network is two-fold: that currently useless single-field fundus photographs can be used for disease staging of diabetic retinopathy and that screening of fundus photographs is facilitated.

The convolutional neural network GoogLeNet was created for the general image classification of 256 × 256 size images[10] but the network is thought to be useful for only four classes with large images, such as 1,272 × 1,272 pixels, which we used in the first experiment. In our preliminary experiments, AlexNet[9] did not achieve high accuracy, and ResNet[11] could not store such large images in the 12 GB graphical processing unit memory. It was thus restricted to 256 × 256 size images and did not achieve high accuracy (data not shown).

Surface reflection of the retina was enhanced in 2 of the 20 PDR images (Fig 4F). Reflection has not previously been reported as a criterion of DMR. Surface reflection of the retina is thought to be influenced by thickening of the internal limiting membrane, which has already been reported in PDR but not used in grading.[23] Although surface reflection is often seen in very young people, it is clear and not coarse. It is difficult to use reflection as a criterion of DMR because of the difficulty of separating clear and coarse reflections. Nonetheless, these findings suggest that deep learning might be a useful detector of novel classification criteria.

In the second experiment, it was difficult for the human judges to grade the images from 0 to 14 because there were no clear criteria. A lack of clear criteria is not a problem for machine learning. Most patients with PDR did not need treatment (Table 2) because the eyes that had previously undergone panfundus photocoagulation were graded as having PDR.

Advances in deep learning have improved AI.[9–11] In medicine, however, AI is mostly used as an alternative to human graders in the field of diabetic retinopathy[24] for detecting retinal hemorrhage[25] or classifying single photographs.[27] Experienced ophthalmologists have gathered impressions of patients’ prognoses, but these impressions are not quantitative. Therefore, we trained a deep convolutional neural network using single posterior pole color fundus photographs of many eyes to determine staging involving parts of the retina not visible in the photograph. In addition, the neural network was trained similarly to also determine prognosis. This AI grading system can be applied to other diseases and may improve prognosis.

This study has some limitations. First, it was based on conventional one-field 45° fundus photographs. Machine learning that can be trained on ultra-widefield 200° scanning laser ophthalmoscopy[17] is needed. Second, the modified Davis grading is not as common. Thus, AI trained by other common grading methods is needed. Third, for disease staging, the significance of false predictions often differs according to class. For example, if a healthy patient (NDR) is wrongly predicted as having PDR (false positive), the consequences are not particularly grave because the physician will realize the mistake. However, if a PDR patient is wrongly predicted as being NDR (false negative), there might be severe consequences because the lack of follow-up treatment may lead to health problems. In this study, the false negative rate was lower than the false positive rate but was still 12%. AI that considers the importance of false negative findings is also needed.

## Conclusion

We proposed a novel AI disease-staging system that grades diabetic retinopathy using a retinal area that is not usually visualized on fundoscopy and another AI that directly suggests treatments and determines prognoses.
